# Supplementary material for: A Single Nucleotide Mutation in Adenylate Cyclase Affects Vegetative Growth, Sclerotial Formation and Virulence of Botrytis cinerea
Source: Int J Mol Sci. 2020 Apr 21;21(8):2912. doi: 10.3390/ijms21082912 (PMC7215688; doi:10.3390/ijms21082912)
Supplement: Supplementary file 1 [file ijms-21-02912-s001.zip › ijms-770707-supplementary/supplementary/Figure S1-S4.docx]

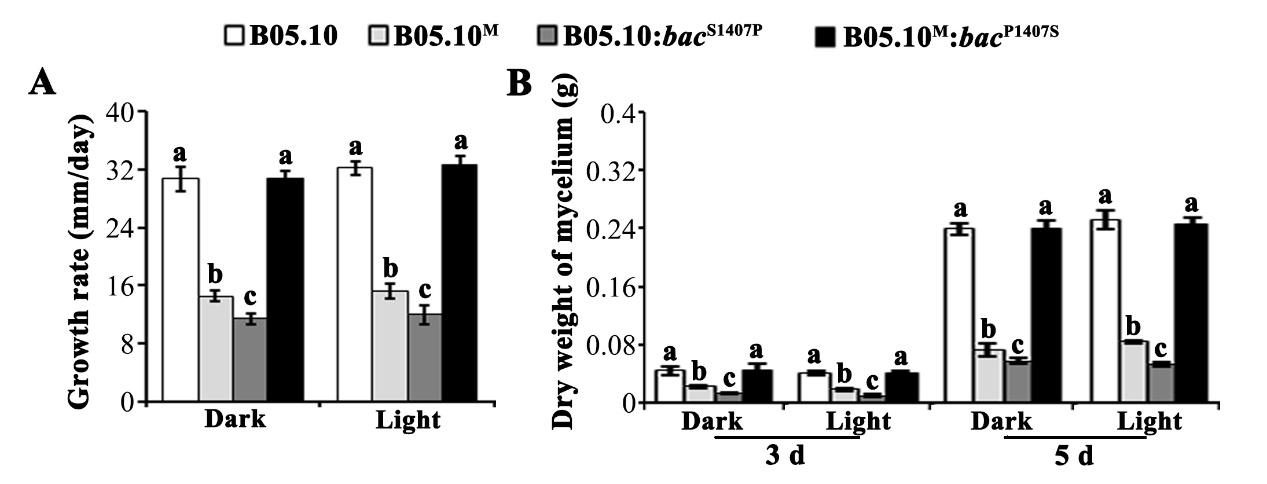


**Figure S1.** The S1407P mutation in *bac* resulted in decreased mycelial growth rate and decreased biomass accumulation. (A) Mycelial growth rates of B05.10, B05.10^M^, B05.10:*bac*^S1407P^and B05.10^M^:*bac*^P1407S^ in dark and light on CM. The growth rate of each strain was determined from the colony diameter on the second day relative to that on the first day after incubation. (B) Mycelial dry weights of the above strains on CM under dark and light for 3 and 5 days. Different letters on the columns indicate significant differences (P < 0.05).


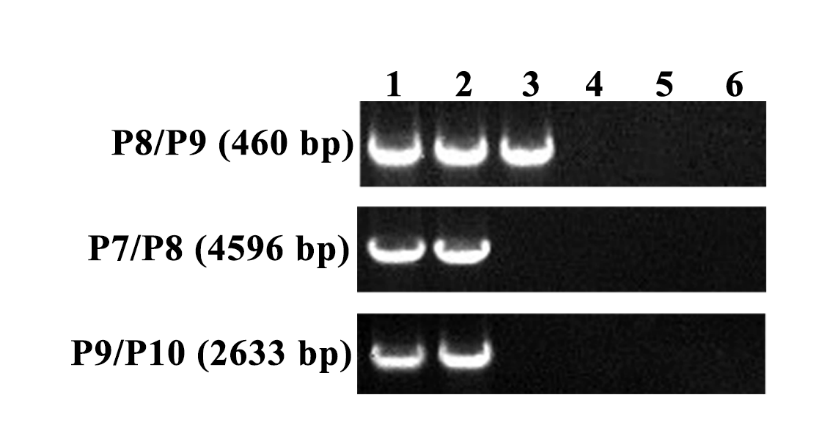


**Figure S2.** Identification of point mutants by PCR. Panels 1-6 show B05.10:*bac*^S1407P^, B05.10^M^:*bac*^P1407S^, pNAN-OGG plasmid, B05.10, B05.10^M^ and negative control (water), respectively.


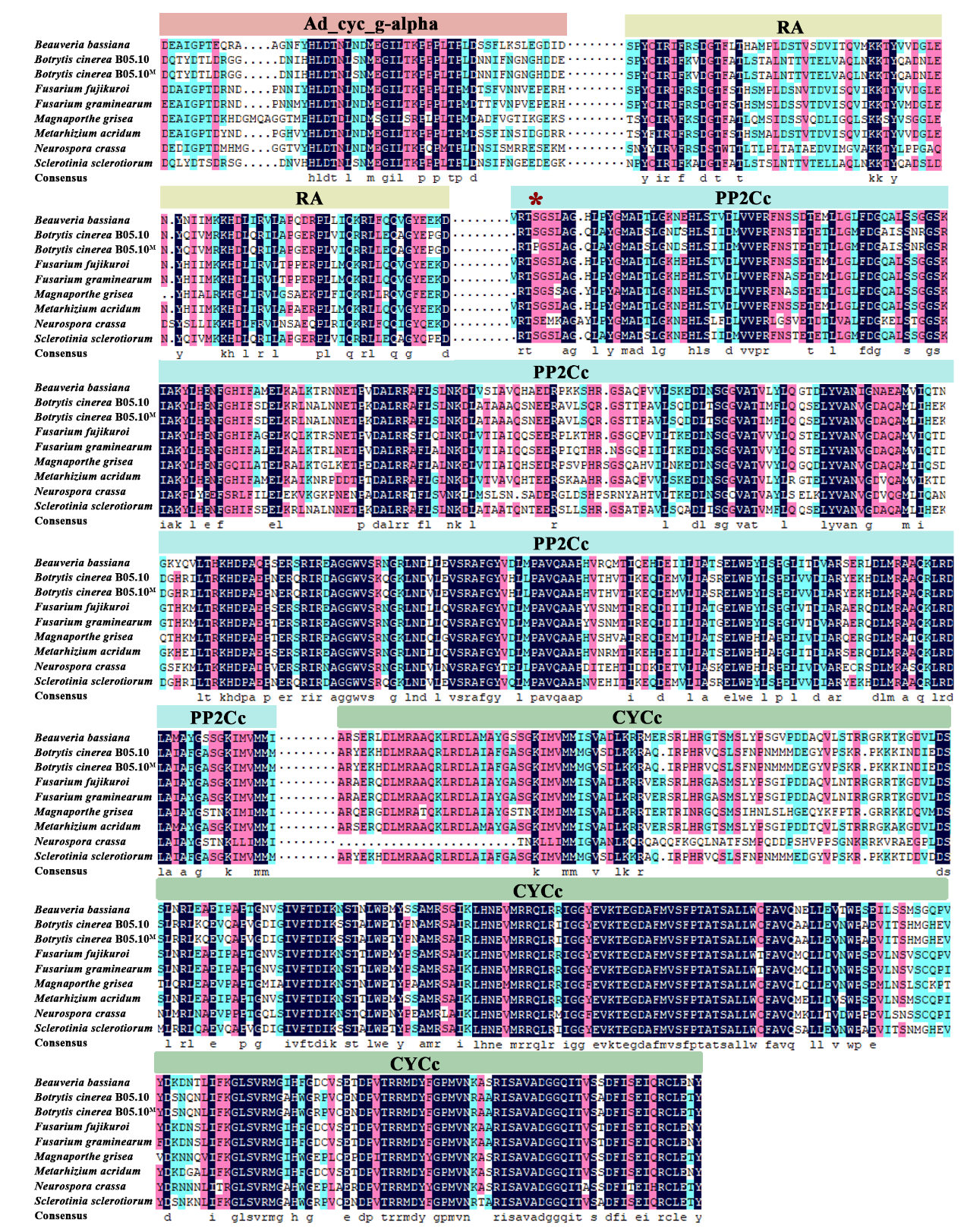


**Figure S3.** Sequence alignment of adenylate cyclase in fungi. The alignment was generated using DNAMAN. Ad_cyc_g-alpha: G-alpha binding domain; RA: Ras association domain; PP2Cc: PP2Cc--type phosphatase domain; CYCc: AC catalytic enzyme domain. The asterisk (*) in PP2Cc domain represents the mutation site (S1407P).


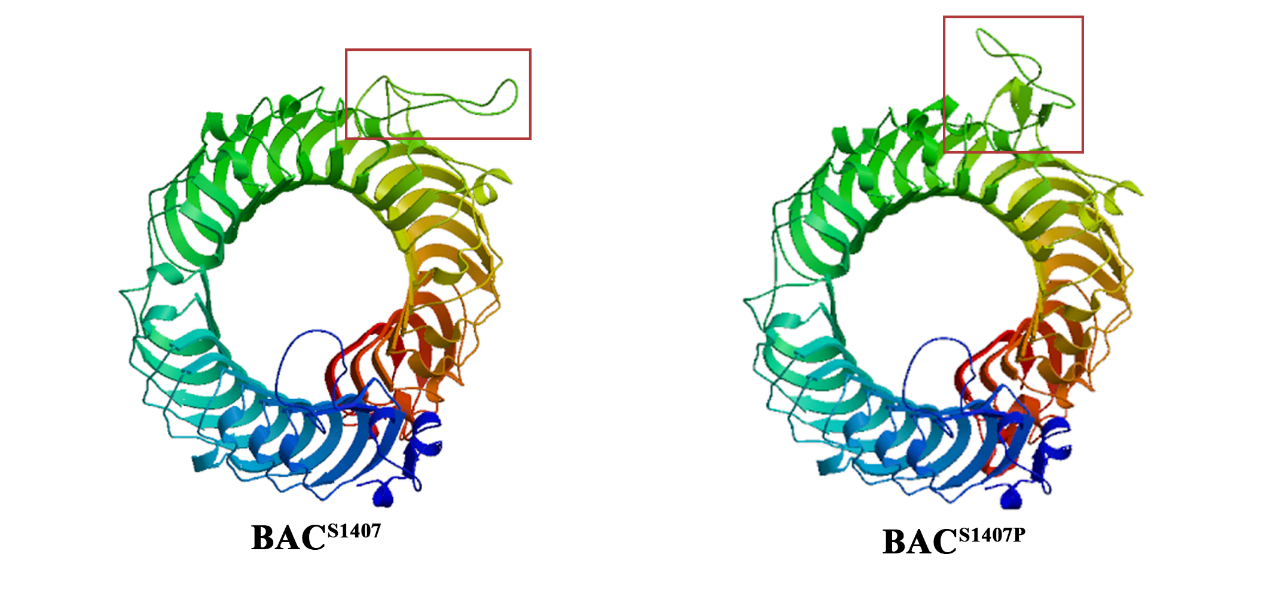


**Figure S4.** The protein structure prediction of wild type BAC^S1407^ and the point mutation BAC^S1407P^.
