# Supplementary material for: A Single Nucleotide Mutation in Adenylate Cyclase Affects Vegetative Growth, Sclerotial Formation and Virulence of Botrytis cinerea
Source: Int J Mol Sci. 2020 Apr 21;21(8):2912. doi: 10.3390/ijms21082912 (PMC7215688; doi:10.3390/ijms21082912)
Supplement: Supplementary file 1 [file ijms-21-02912-s001.zip › ijms-770707-supplementary/supplementary/Table S1.docx]

**Table S1.** Colony diameters, conidiation and sclerotia formation of different strains in dark and light.

| **strain** | **Colony diameters (mm)** | | **Conidiation (×10^7^)** | | **Sclerotia formation** | |
| --- | --- | --- | --- | --- | --- | --- |
|  | Dark | Light | Dark | Light | Dark | Light |
| B05.10 | 78.19±0.46 | 79.75±0.08 | 0 | 22.60±3.83 | 204.33±28.57 | 0 |
| B05.10^M^ | 46.22±0.93 | 49.63±1.29 | 2.66±0.31 | 9.06±0.69 | 0 | 0 |
| B05.10:*bac*^S1407P^ | 39.95±0.91 | 44.19±1.34 | 3.96±0.61 | 9.84±0.26 | 0 | 0 |
| B05.10^M^:*bac*^P1407S^ | 78.77±0.69 | 79.83±0.15 | 0 | 21.25±2.70 | 212.33±25.81 | 0 |
