# Supplementary material for: A Single Nucleotide Mutation in Adenylate Cyclase Affects Vegetative Growth, Sclerotial Formation and Virulence of Botrytis cinerea
Source: Int J Mol Sci. 2020 Apr 21;21(8):2912. doi: 10.3390/ijms21082912 (PMC7215688; doi:10.3390/ijms21082912)
Supplement: Supplementary file 1 [file ijms-21-02912-s001.zip › ijms-770707-supplementary/supplementary/Table S3.docx]

**Table S3** Oligonucleotide primers used for target amplification in this study

| Primer | Nucleotide sequence (5’-3’) | Target of amplification |
| --- | --- | --- |
| P1 | TTAAGAAGGAGCCCTTCACCACTAGTAAGCACTTCCGCAAGTACAATTAAT | Primers to amplify 5’ region fragment for construction of *bac* point mutation vector |
| P2 | AAAAATGCTCCTTCAATGTCCTCATACATATGGTGCAGAA |  |
| P3 | TTCTGCACCATATGTATGAGGACATTGAAGGAGCATTTTT | Primers to amplify resistance cassette fragment for construction of *bac* point mutation vector |
| P4 | GACGCAGTCTTTATGATGATGCATTGGATTAATAATTGTTG |  |
| P5 | CAACAATTATTAATCCAATGCATCATCATAAAGACTGCGTC | Primers to amplify 3’ region fragment for construction of *bac* point mutation vector |
| P6 | GGGTCGGCGCGCCCACCCTTACTAGT CGGGAGACGATGTGATGC |  |
| P7 | TTCGGTCTCCGTCTGCGAATG | Primers to identify *bac* site-directed mutant |
| P8 | AAGACGGTGTCGGTGGTG |  |
| P9 | GACATTGAAGGAGCATTTTTTGGGC | Primers to identify *bac* site-directed mutant |
| P10 | CAGGCAACCAGGAGTGAA |  |
