# Supplementary figures and images for: A Single Nucleotide Mutation in Adenylate Cyclase Affects Vegetative Growth, Sclerotial Formation and Virulence of Botrytis cinerea
Source: Int J Mol Sci. 2020 Apr 21;21(8):2912. doi: 10.3390/ijms21082912 (PMC7215688; doi:10.3390/ijms21082912)

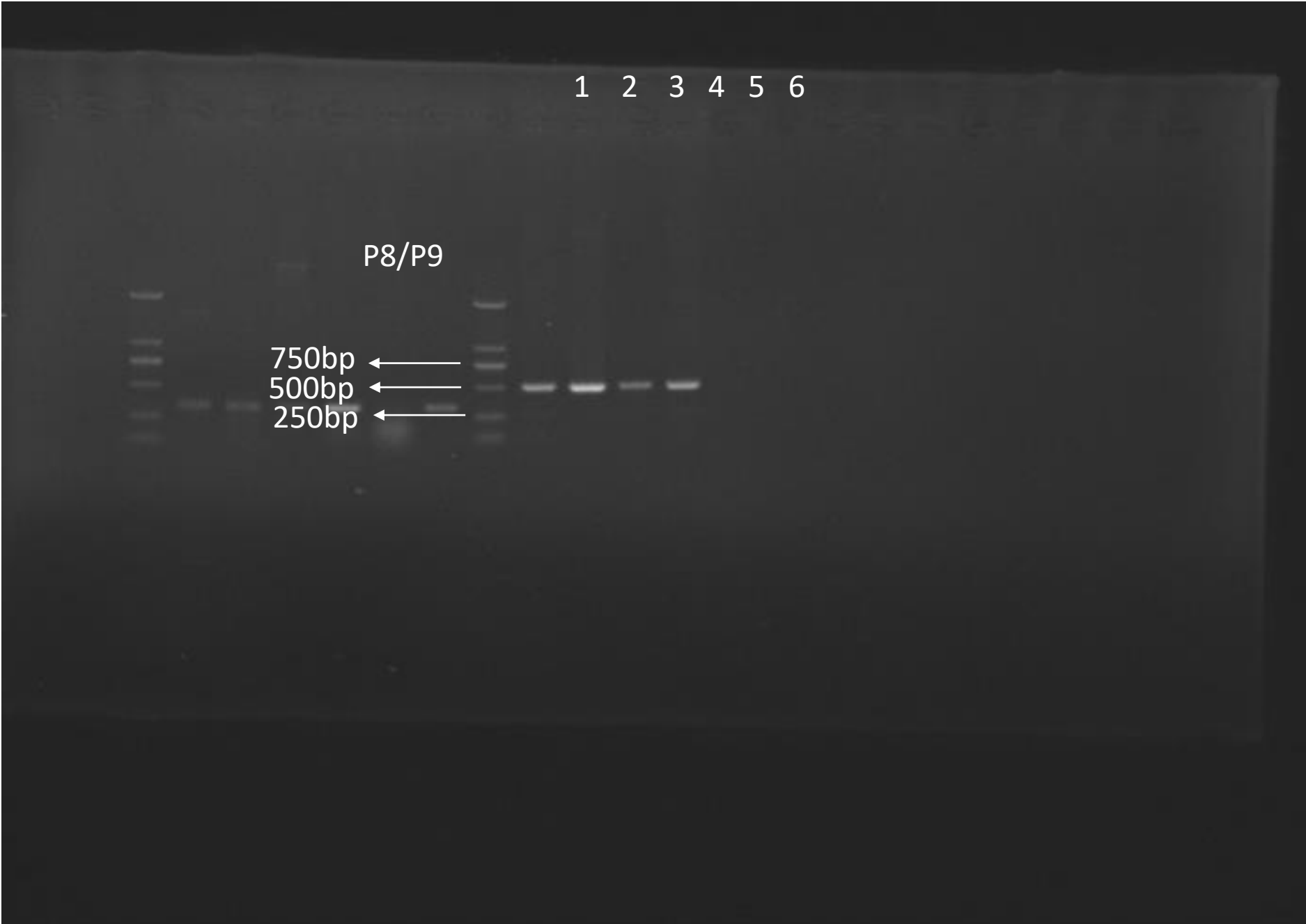

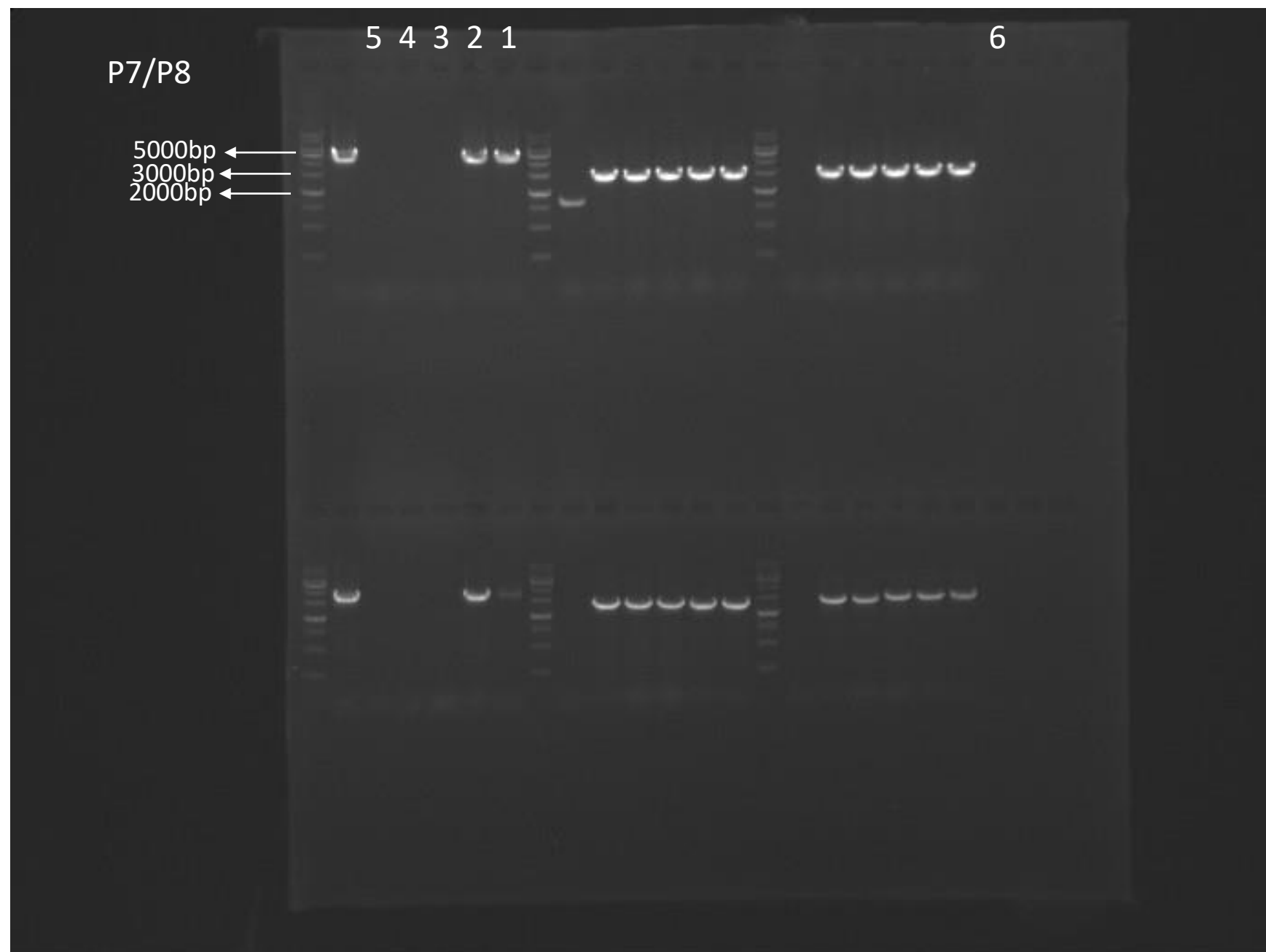

P9/P10

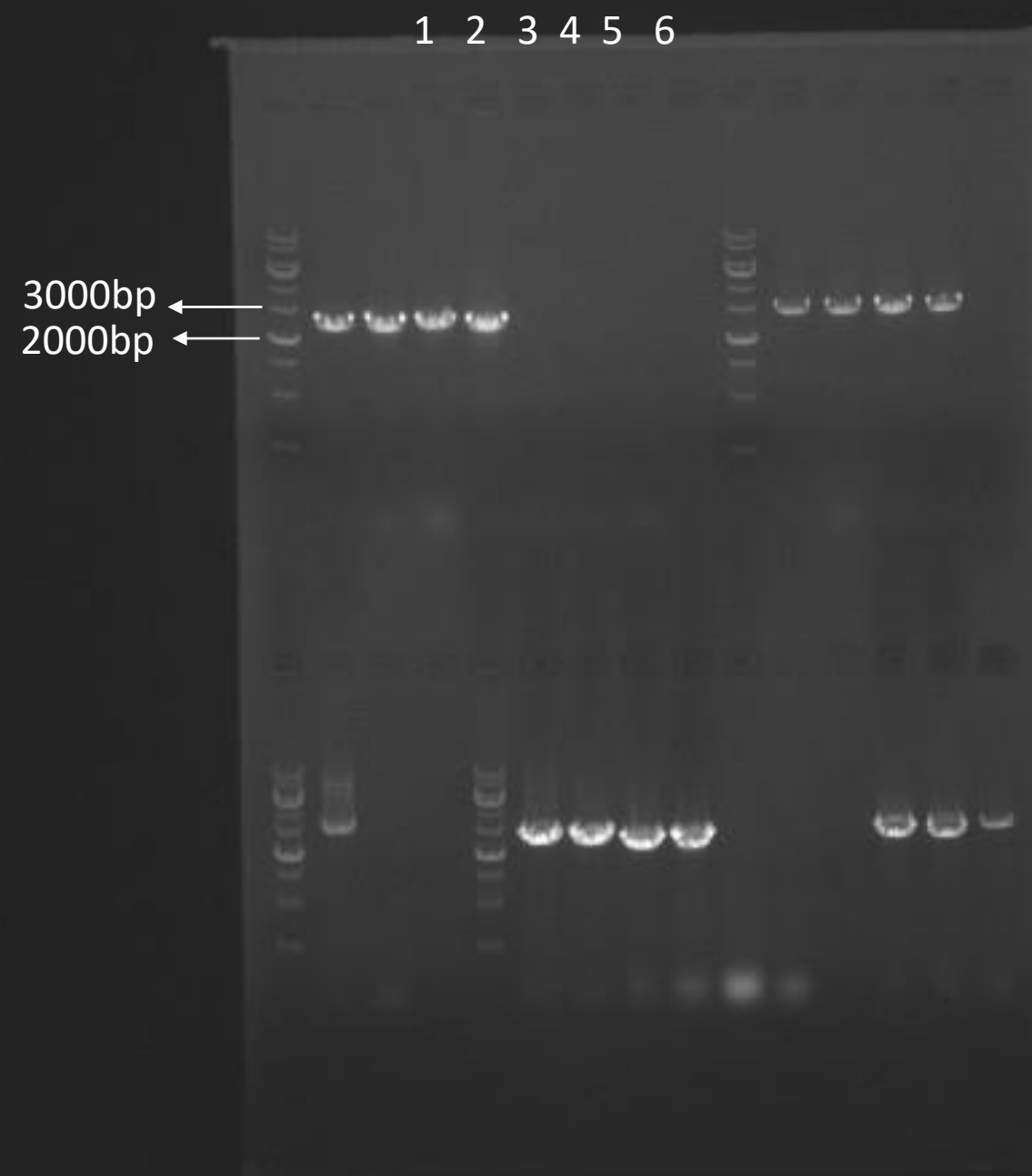

Supplement: Supplementary file 1 [file ijms-21-02912-s001.zip › ijms-770707-supplementary/supplementary/Figure S2 PCR Gel picture.pdf]
